# Navigating Virology’s Frontiers in Africa: Global Virus Network 2024 Durban Meeting

**DOI:** 10.3390/v17060819

**Published:** 2025-06-05

**Authors:** Maggie L. Bartlett, Rubeshan Perumal, Sten H. Vermund, Salim Abdool Karim

**Affiliations:** 1 Global Virus Network, Tampa, FL 33612, USA; rubeshan.perumal@caprisa.org (R.P.); svermund@gvn.org (S.H.V.); salim.abdoolkarim@caprisa.org (S.A.K.); 2 Johns Hopkins Bloomberg School of Public Health, Baltimore, MD 21205, USA; 3 Centre for the AIDS Programme of Research in South Africa (CAPRISA), South African Medical Research Council (SAMRC)-CAPRISA-TB-HIV Pathogenesis and Treatment Research Unit, University of KwaZulu-Natal Nelson R Mandela School of Medicine, Durban 4001, South Africa; 4 College of Public Health, University of South Florida, Tampa, FL 33620, USA; 5 Department of Epidemiology, Mailman School of Public Health, Columbia University, New York, NY 10027, USA

**Keywords:** virology, Africa, pandemic, scientific misinformation, zoonosis

## Abstract

The Global Virus Network (GVN) is a voluntary consortium of virology laboratories and affiliated scientists that seek to prevent and control global viral threats. The meetings of the GVN are characterized by academic, health center, government, and industry participation, sharing information that is designed to further the mutual mission. In September 2024, the meeting in Durban, South Africa, highlighted diseases and investigators from Africa, and paid special attention to pandemic preparedness. Selected highlights from the meeting are presented here, along with a call-to-action in defense of global partnerships for research in the origins of human and animal viruses, the risk to humans from other animal sources, the pathogenesis of given viruses, and their prevention and treatment. Discussions of laboratory discovery science are juxtaposed with development of vaccines, antiviral drugs, immunotherapies, and innovative field strategies for control of viral diseases.

## 1. Introduction

**Figure 1:**
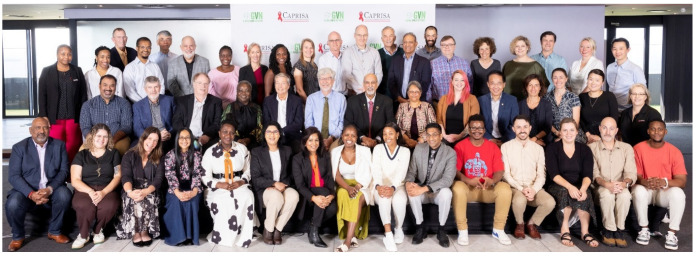
Participants of GVN “Navigating Virology’s Frontiers in Africa” Annual Meeting.

With the goal of amplifying expertise and collaboration globally in virology, the Global Virus Network (GVN) was formed in 2011. The consortium sought to provide virologists with the opportunity to connect, collaborate, and elevate the discipline globally. The GVN hosts a global meeting at one of its ≈85 affiliated Centers of Excellence or Affiliates, selecting the Centre for the AIDS Programme of Research in South Africa (CAPRISA) in Durban as the meeting host on 16 to 18 September 2024 (Figure 1).

The GVN Durban meeting highlighted critical advancements in pathogen detection, immune responses, and pandemic preparedness, while underscoring the necessity of fostering the next generation of virologists and fighting misinformation. Held in Africa, the event emphasized the unique challenges and opportunities that the continent faces, from understanding regional viral variants to addressing disparities in healthcare access. The meeting showcased Africa’s growing role in global health, aiming to enhance collaboration, build local research capacity, and ensure that pandemic solutions are inclusive and equitable. Here, we summarize highlights of selected presentations.

## 2. Evading Viral Evasion

Dr. Alan Landay (University of Texas Medical Branch) emphasized the growing necessity for more reliable, integrated, and objective methods to detect pathogens and address the complexity of host responses. He discussed the critical role of bioinformatics and analytic tools in pathogen discovery, particularly in the context of HIV, SARS-CoV-2, and Crimean-Congo hemorrhagic fever virus. Dr. Landay highlighted the uniqueness of biological aging across different organs, pointing to accelerated aging markers in blood from individuals on antiretroviral therapy, where oxidative and glycolytic pathway imbalances play a central role [1,2].

Dr. Sarah Londrigan (The Peter Doherty Institute for Infection and Immunity) delved into the role of macrophages within the airway immune system, particularly in relation to influenza A virus (IAV) infections. Her findings reveal that IAV’s infection in macrophages is abortive, where viral ribonucleoproteins (vRNPs) are crucial, yet the macrophages act as a “dead end” for viral propagation [3]. Londgrin’s research showed that red blood cells (RBCs) could effectively deplete viral loads due to their glycan-binding capabilities. Her insights on the airway microenvironment offer a novel understanding of how macrophages regulate immune responses to seasonal IAV.

Dr. Vineet Menachery (University of Texas Medical Branch) discussed defective interfering RNA and its often-overlooked role in viral recombination. His team found SARS-CoV-2 to be significantly more recombinogenic than the common cold or MERS. Notably, they discovered microdeletions near UUAU sites that enhance viral recombination, driven by the action of viral non-structural protein 15 (NSP15) [4]. Blocking NSP15 led to reduced infection but did not mitigate the associated pathology. Instead, recombination increased, suggesting that NSP15 may serve as a complex regulatory target in SARS-CoV-2 infections.

## 3. Translational Virology and Complex Co-Infections

Dr. Kizzmekia Corbett-Helaire (Harvard T.H. Chan School of Public Health) discussed how the immune landscape of the coronavirus spike is shaped by the receptor binding domain (RBD), the primary target for neutralizing antibodies, while the conserved N-terminal domain (NTD) provides protection when RBD immunity wanes. Immunized mice survived lethal MERS-CoV exposure despite low neutralizing antibody levels. Novel nanoparticle designs aim to enhance B-cell engagement and responses to hidden neutralizing epitopes using mRNA or DNA delivery. Cross-reactivity studies indicated that MERS nanoparticles elicited stronger binding responses to SARS-CoV-1 and WIV-1 spikes, informing universal coronavirus vaccine design [5].

Dr. Peter Quashie (University of Ghana) presented an analysis of the COVID-19 pandemic in Ghana, where over 171,000 cases resulted in a 1% death rate. His research challenged assumptions about the mildness of COVID-19 in West Africa and investigated whether cross-protection from malaria or other endemic infections played a role in the region’s immune response [6]. Previous work in 2022 suggested that 68% of viral genomes in Ghana were variants of concern (VOC) [7]. The presentation explored the immune implications of prior viral exposures in shaping the region’s response to COVID-19 and how to address systemic errors in viral phylogenies [8].

Dr. Susan Weiss (University of Pennsylvania) reported on the zoonotic transmission of MERS-CoV, which begins with *Neoromicia capensis* bats and spreads to camels before infecting humans. While camels experience only mild symptoms, human infections can be severe and result in limited human-to-human transmission. Dr. Weiss investigated viral replication in human nasal and bronchial cells, finding that MERS replication is restricted in nasal cells, likely due to receptor limitations, providing insights into the viral behavior at early infection stages [9].

## 4. Acute and Post-Viral Diseases

Dr. Maggie Bartlett (Johns Hopkins Bloomberg School of Public Health) gave an overview of dysautonomia, and neurotropic viruses included many viruses that cause post-acute infectious syndrome (PAIS), focusing on transcriptomics of neurons and how viruses alter messaging within infected cells leading to noncytolytic clearance. Preliminary clinical data were shared on viral recrudescence following treatments for PAIS and plans for continued work elucidating differences in those with hereditary connective tissue issues and their predisposition to PAIS [10].

Dr. Marc Lecuit (Institut Pasteur) discussed those pathogens with the ability to cause maternal–fetal infections and infections of the central nervous system. Dr. Lecuit shared data that compared the gut microbiota of children with Japanese encephalitis virus (JEV) in Southeast Asia. Lower gut microbiota diversity was observed in JEV cases, which could lead to reduced interferon signaling and immature immune responses.

Dr. Alfredo Garzino-Demo (University of Maryland, Baltimore) examined the microRNA differences in olfactory cells to probe the anosmia caused by SARS-CoV-2. RNA-seq data showed clear separation between the groups. Between those with persistent olfactory symptoms and those with persistent non-olfactory symptoms, there were differentially expressed genes involved in neutrophil activation, which aligns with the current knowledge of SARS-CoV-2 pathogenesis. In the cohort with persistent olfactory symptoms, genes involved in inflammatory signaling were upregulated compared to controls. Between those with olfactory symptoms and those with no olfactory symptoms, there was enrichment in metallothionein genes. Metallothionein binds zinc, sequestering it, which may lead to lower available zinc levels.

## 5. Bats, Rats, and Other Vectors

Dr. Linfa Wang **(Duke-NUS Medical School)** opened the session by discussing the unique biology of bats and their ability to serve as reservoirs for a variety of pathogens. He emphasized the concept of “anti-disease” vs. “anti-pathogen” approaches, focusing on host immunity adaptation rather than solely targeting pathogens. Bats’ unique biology allows them to tolerate viral infections without succumbing to disease, making them a key focus for future biomedical research. One of his groundbreaking discoveries involved the *ASC2* gene in bats, which plays a critical role in inflammation control. Dr. Wang’s team developed transgenic mice expressing bat *ASC2*, which demonstrated protection against influenza A virus, reducing lung inflammation without affecting viral load [11].

Dr. Jonathan Towner (**US CDC**) presented his work on tracking the movements of bat reservoirs, specifically focusing on the Marburg virus, a filovirus that causes hemorrhagic fever. Dr. Towner and his team utilized micro-GPS devices to monitor the nocturnal movements of Marburg virus-positive bats in Uganda. His research revealed the large distances bats can travel—up to 49.5 km in a single night—and their preference for specific habitats, such as fruit-bearing trees near human settlements.

Dr. Adeola Fowotade (University of Ibadan, Nigeria) provided a clinical perspective from Nigeria, Africa’s most populous country. Her two case studies of viral infections and genomic analyses show that viruses belonged to clade IIb but represented three distinct lineages. Fowotade highlighted that lineage A, endemic to Nigeria, has been circulating between 2017 and 2022 through sustained human-to-human transmission, while lineage B is the strain circulating globally. Her research underscores the importance of genomic surveillance in tracking viral evolution and transmission patterns.

## 6. Genetic Sequences and Epidemiological Insights

Dr. Laura Dickson (University of Texas Medical Branch) highlighted how climate change is reshaping arbovirus transmission, particularly through *Aedes aegypti* adaptation. Urbanization and drier environments increase Zika virus (ZIKV) susceptibility in *A. aegypti*, yet most temperature-based models fail to incorporate humidity effects. Lower humidity reduces mosquito survival but paradoxically increases blood-feeding rates, potentially enhancing virus transmission [12].

Dr. William de Souza (University of Kentucky) reported on the Oropouche virus, a midge-borne orthobunyavirus responsible for febrile illness, CNS infections, and pregnancy complications [13]. Its increasing spread in South America underscores the need for enhanced surveillance and expanded development and deployment of affordable diagnostics.

Dr. Scott Weaver (University of Texas Medical Branch) discussed recent findings on viral evolution and cross-immunity. While Oropouche virus traditionally has a low case fatality rate, new data suggest recent increases, surpassing dengue in some regions. He also explored Western equine encephalitis virus (WEEV) evolution in North America. Notably, chikungunya virus (CHIKV) immunity appears to reduce susceptibility to Mayaro virus, suggesting that CHIKV circulation could help prevent future Mayaro outbreaks [13,14].

## 7. Cutting-Edge Diagnostics and Therapeutics

Dr. Marc Bonneville (Instititut Merieux) emphasized the need for advancing multiplex diagnostic platforms to simplify pathogen detection for clinicians. He discussed the importance of real-time surveillance, particularly in high-burden areas like sub-Saharan Africa, where automated diagnostic platforms could significantly improve public health outcomes by allowing rapid response to emerging pathogens [15].

Dr. Anne Wyllie (Yale University) emphasized saliva as a cost-effective sample type for outbreak control. Early saliva collection devices ranged from USD 7 to USD 28, with higher costs linked to stabilizing buffers, which her research found unnecessary. Studies showed stable SARS-CoV-2 detection in saliva under various conditions, including storage at 30–40 °C and mail transit for 56 h [16]. Similar results were found for influenza A/B, RSV, and Mpox, supporting saliva’s potential for remote, cold chain-free testing. Saliva can detect SARS-CoV-2 and Mpox earlier and at higher viral loads than nasal swabs, making it ideal for pre- and asymptomatic screening.

Dr. Nokukhanya Msomi (CAPRISA) discussed current and emerging strategies for hepatitis B treatment, emphasizing viral suppression and the shift toward a functional cure—sustained HBV DNA suppression off treatment, HBsAg loss, and seroconversion. A major challenge is the persistence of covalently closed circular DNA (cccDNA), which drives chronic infection. New therapies aim to silence cccDNA, prevent transcription, and enhance viral decay. Diagnostic tools are evolving to measure ultra-low levels of HBsAg and HBV DNA, with additional biomarkers like core-related antigen and HBV RNA under investigation. A multi-target approach, similar to HIV treatment, may improve outcomes by combining nucleotide analogs, interferons, and novel antivirals.

## 8. Pandemic Preparedness

Dr. Jana Broadhurst (University of Nebraska Medical Center) underscored the need for community trust to be at the center of pandemic response. Pandemics continually face challenges in diagnostic testing, beginning with limited tools and struggling to rapidly develop novel solutions. A critical issue is the lack of coordination among clinical, public health, and research sectors in defining prioritized test use cases. While there is a strong effort in generating target profiles for diagnostics, improved collaboration is essential. Scaling up diagnostic responses remains slow, as seen in COVID-19, and requires community trust and engagement to ensure accessibility. Community-based approaches, such as saliva sampling, have shown success in remote Nebraska, where self-collected specimens maintained integrity and enabled variant sequencing of nearly 80% of positive SARS-CoV-2 cases.

Dr. Rachel Roper (East Carolina University) discussed the history of poxviruses and provided an overview of the status today. Mpox often presents with a single lesion, leading to missed diagnoses. The 2003 outbreak was linked to infected prairie dogs that were co-housed with imported African rodents (African giant pouched rats, dormice, and rope squirrels) for the pet trade. In 2023-2024, cases surged globally, with WHO reporting 97,000 cases and 200 deaths by May 2024. Underreporting due to inadequate surveillance and testing suggests higher true case numbers. The emergence of Clade 1, a more virulent strain with human-to-human transmission, raises concerns. While Clade 1 cases have declined in Europe and the Americas, they are rising in Africa and the Western Pacific. Safe poxvirus-based vaccines like MVA remain crucial in mitigating Mpox’s impact.

Dr. Alash’le Abimiku (Institute of Human Virology, Nigeria) highlighted the role that data science plays in pandemic preparedness. Genomic analyses from GISAID revealed that early pandemic waves were underreported due to limited sequencing capacity. The Social Vulnerability Index (SVI) quantifies a community’s resilience, with findings indicating that high-income individuals reduced travel distances but not frequency during lockdowns. Excess COVID-19 deaths disproportionately affected HIV-positive individuals and those with metabolic diseases; however, vaccines provided substantial protection, even among immunocompromised populations, underscoring the need for equitable vaccine distribution.

## 9. Scientific Misinformation and Public Trust

The session on scientific misinformation, led by Dr. Salim Abdool Karim, focused on the global challenge of combating misinformation during pandemics and outbreaks [17]. The discussion addressed the complexities of conveying accurate scientific information to the public in an age dominated by social media, where misinformation spreads rapidly. Dr. Abdool Karim emphasized the importance of clear communication strategies, transparency from public health officials, and robust fact-checking mechanisms. Panelists discussed the psychological factors that make individuals more susceptible to misinformation and highlighted the importance of training scientists and public health professionals in effective communication. The session underscored that restoring public trust in science requires sustained efforts from both the scientific community and media outlets to counteract misinformation and promote health literacy. This is especially compelling when arguing for donor nation assistance to tackle emerging infections at their sources in low- and middle-income countries [18,19].

## 10. Conclusions

The 2024 GVN Annual Meeting in Durban served as a testament to the power of global scientific collaboration in virology. By bringing together leading experts, emerging researchers, and key stakeholders, the conference fostered critical discussions on pathogen surveillance, vaccine development, and innovative strategies for pandemic preparedness. The emphasis on Africa’s role in global virology highlighted the region’s scientific contributions and underscored the importance of equitable access to research funding, infrastructure, and training. As misinformation continues to challenge public health responses, the meeting reinforced the need for science-driven policies and community engagement. Looking ahead, the GVN remains committed to strengthening international partnerships, advancing cutting-edge virology research, and ensuring that solutions to global health threats are both inclusive and sustainable.
